# The Effect of Topology on Block Copolymer Nanoparticles: Linear versus Star Block Copolymers in Toluene

**DOI:** 10.3390/polym14173691

**Published:** 2022-09-05

**Authors:** Yuan Zhang, Peng Wang, Nan Li, Chunyan Guo, Sumin Li

**Affiliations:** Research School of Polymeric Materials, School of Materials Sciences & Engineering, Jiangsu University, Zhenjiang 212013, China

**Keywords:** topology, star-block copolymer, polymerization-induced self-assembly, general self-assembly

## Abstract

Linear and star block copolymer (BCP) nanoparticles of (polystyrene-*block*-poly(4-vinylpyridine))_n_ (PS-*b*-P4VP)_n_ with arm numbers of 1, 2, 3, and 4 were prepared by two methods of polymerization-induced self-assembly (PISA) and general self-assembly of block copolymers in the low-polar organic solvent, toluene. The effect of the arm number on the size and/or morphology of the (PS-*b*-P4VP)_n_ nanoassemblies synthesized by the two methods in toluene and on the polymerization kinetics was investigated in detail. Our results show that in toluene, a low-polar solvent, the topology not only affected the morphology of the BCP nanoparticles prepared by PISA, but also influenced the BCP nanoparticles synthesized through general self-assembly.

## 1. Introduction

Block copolymer (BCP) self-assembly has attracted much attention in the past several decades for the synthesis of polymeric nano-objects of various morphologies and their potential applications in many fields [1,2]. In general, two strategies, micellization of amphiphilic block copolymers in block-selective solvent [3,4,5,6,7,8,9,10] and polymerization-induced self-assembly (PISA) [11,12,13,14,15,16,17,18,19], have been adopted to synthesize/prepare BCP nano-assemblies. In the first strategy, pre-synthesized amphiphilic block copolymers are initially dissolved in a common solvent [3,4,5] and then block-selective solvent is added to induce the soluble-to-insoluble phase transition of the solvophobic block and therefore trigger micellization of the amphiphilic block copolymers in the solvent [6,7,8,9,10]. In the PISA strategy, all ingredients including a monomer, macromolecular chain transfer agent, and initiator are dissolved in solvent in the initial polymerization stage [11,12,13]. With the proceeding of the polymerization, the newly formed hydrophobic chain grows to a certain length, and in situ self-assembly of amphiphilic block copolymers occurs and micelles are formed [14,15,16]. After that, dispersion polymerization takes place dominantly in the monomer-swollen micelles, resulting in the change in size or morphology of the block copolymer nanoparticles [17,18,19]. Therefore, the synthesis and assembly of amphiphilic block copolymers are carried out simultaneously in one pot in the PISA method [11,12,13,14,15,16,17,18,19].

It is well known that the morphologies of amphiphilic BCPs are dependent on the solvent characteristics [20,21] and especially the intrinsic molecular architecture [22,23,24,25,26,27]. Star block copolymers include a single branch point from which chemically different building blocks spread out [28,29,30,31]. Owing to this exclusive structure, star block copolymers show many interesting characteristics and properties unattainable by linear polymers [22,23,24,25,26,27]. For example, Ma and coworkers successfully synthesized the novel dual-functional linear and star POSS-containing organic–inorganic hybrid block copolymer poly(glycidyl methacrylate)-*block*-poly(methacrylisobutyl polyhedral oligomeric silsesquioxane)_1,4,6_ ((PGMA-*b*-PMAPOSS)_1,4,6_) by the core-first ATRP method, then crosslinked the block copolymers in the presence of trimethylamine to form a 3-dimensional network. Furthermore, the self-assembly behavior of these block copolymers with similar volume fractions of insoluble block was investigated in detail in water. It was revealed that the linear block copolymers (BCPs) PGMA-*b*-PMAPOSS showed the formation of spherical micelles with a size of 140–200 nm, star four-arm BCPs (PGMA-*b*-PMAPOSS)_4_ assembled as a multi-core core-shell morphology, and star six-arm BCPs (PGMA-*b*-PMAPOSS)_6_ assembled as the dendritic feature [26]. Huh and his team found that self-assembling the amphiphilic star poly(ethylene glycol)-[poly(ε-caprolactone)]_2_ and the corresponding linear counterparts with approximately the same chemical composition in selective solvent lead to cylindrical and spherical micelles, respectively [27].

The preparation of star block copolymer self-assemblies through the first strategy, that is, general self-assembly, has been widely reported [9,10]. In addition, there are also few reports on the preparation of star block copolymer assemblies via the PISA method, and most of them are carried out in a polar solvent, such as water or ethanol/water mixture solution [32,33,34,35,36,37]. As we all know, solvent properties or composition have an impact on the morphology of block copolymer nano-assemblies [20,21]. In addition, BCP nanoparticles prepared in low polar solvents could be easily used as lubrication and emulsifiers for water/oil emulsions [38,39]. However, rare reports on the effect of the arm number on block copolymer nano-assemblies, i.e., linear block copolymers versus star block copolymers in low polar organic solvent, have been reported.

The polystyrene-mediated RAFT dispersion polymerization of the 4-vinylpyridine (4VP) monomer is a typical example that uses the low-polar solvent, toluene [40,41]. In this paper, the effects of the topological structure on the morphology and evolution process of block copolymer nanoassemblies in toluene were studied using RAFT dispersion polymerization of 4VP regulated by linear or star PS macro-RAFT agents. Firstly, block copolymer nanoassemblies of (polystyrene-*block*-poly(4-vinylpyridine))_n_ ((PS-*b*-P4VP)_n_, n = 1, 2, 3, and 4) were formed through RAFT dispersion polymerization in toluene employing mono- and multi-functional macromolecular chain transfer agents. Then, the polymerization kinetics of these dispersion RAFT polymerizations were investigated, and the effect of the arm number or the DPs of the PS/P4VP chain on the block copolymer nanoassemblies prepared via PISA was explored by changing the length of PS or P4VP. Finally, 2-, 3-, and 4-arm star and linear block copolymers of (PS-*b*-P4VP)_1,2,3,4_ with similar chemical compositions were selected to prepare the nanoassemblies in toluene by the general self-assembly method to further prove that the topology also has an important effect of on the morphology of block copolymer nanoassemblies prepared by general self-assembly.

## 2. Experimental Section

### 2.1. Materials

Styrene (St, >98%, Shanghai chemical reagent, Shanghai, China) and 4-vinylpyridine (4VP, 96%, Aladdin) were purified under reduced pressure for later use. The synthesis of the RAFT reagent of four small molecules called I, II, III, IV as shown in Scheme S1 was carried out according to the steps in our previous paper [42,43] and the detailed synthesis process and structure characterization are described in the Supporting Information (Scheme S2) and Figure S1. 2,2′-Azobis(2-methylpropionitrile) (AIBN, >99%, Tianjin Chemical Company, Tianjin, China) was recrystallized from ethanol.

### 2.2. Synthesis of Linear and Star macro-CTAs

Linear and star macromolecular chain transfer agents (macro-CTAs) of (PS_m_-TTC)_n_, in which m and n represent the arm number of the PS arms and the DP of each PS arm, with TTC representing the RAFT terminal of trithiocarbonate, were synthesized by solution RAFT polymerization of St monomers employing I-IV as chain transfer agents (CTAs). Here is a typical synthesis of 3-arm (PS_25_-TTC)_3_ under [St]:[III]:[AIBN] = 450:5:3: St (4.000 g, 0.039 mol), III (0.262 g, 0.427 mmol) and AIBN (42.05 mg, 0.2564 mmol) dissolved in 1,4-dioxane (4.000 g) were weighed into a 38 mL Schlenk flask. The mixture was degassed and ran at 70 °C for 32 h. The styrene conversion was determined by ^1^H NMR employing 1,3,5-trioxane as an internal standard as discussed elsewhere. The synthesized (PS_25_-TTC)_3_ was precipitated into ethanol and then dried under vacuum. By changing the [St]_0_:[CTA]_0_:[AIBN]_0_ molar ratio, other (PS_m_-TTC)_n_ macro-CTAs with different arm chain length and arm number n were also synthesized (Table 1).

The styrene monomer, CTA and AIBN were dissolved in toluene to obtain a homogeneous solution with a solid content of 20 wt%, which was subjected to RAFT dispersion polymerization. Taking an example as a typical representative under [4VP]_0_:[(PS_25_-TTC)_3_]_0_:[AIBN]_0_ = 2400:4:3: into a 38 mL Schlenk flask, 4VP (0.300 g, 2.86 mmol), (PS_25_-TTC)_3_ (38.571 mg, 0.0048 mmol) and AIBN (0.59 mg, 0.0036 mmol) dissolved in toluene (1.350 g) were weighed. The mixture was degassed and then polymerization was ran at 70 °C for a given time. The 4-vinylpyridine conversion was determined by ^1^H NMR. To check the resultant block copolymer nanoassemblies, a small drop of the block copolymer dispersion was deposited onto a piece of copper grid, dried at room temperature under vacuum, and then observed by transmission electron microscope (TEM). To collect the block copolymer for GPC analysis and ^1^H NMR analysis, the synthesized (PS-*b*-P4VP)_3_ nanoassemblies were diluted with dichloromethane and precipitated into ethanol, and finally dried at 40 °C under vacuum.

### 2.3. Preparation of the Block Copolymer Micelles through Self-Assembly in Toluene

The above-synthesized linear or star (PS-*b*-P4VP)_n_ (n = 1, 2, 3) block copolymer nanoassemblies were centrifuged and dissolved in dichloromethane (DCM), then precipitated in toluene, centrifuged and washed with ethanol/toluene (*v*/*v* = 1:8) three times, and finally dried at 40 °C under vacuum to obtain the (PS-*b*-P4VP)_n_ (n = 1, 2, 3) block copolymers. The above processes were designed to eliminate the morphology of the block copolymer nanoassemblies obtained in toluene via the PISA method. Then, (PS-*b*-P4VP)_n_ was dissolved in DCM at room temperature to prepare a 0.5 mg/mL solution, adding a given volume of toluene at a rate of 1 drop (1 drop was about 7 μL) every 10 s under stirring. With the addition of toluene, the solution became turbid, indicating the formation of nanoassemblies. Toluene was then added slowly until the concentration of block copolymer nanoassemblies was about 0.2 mg/mL. Finally, the DCM in the solution was removed under vacuum at 25 °C, and a dispersion solution of block copolymers with 0.2 mg/mL concentration was obtained. TEM was used to characterize the morphology of the (PS-*b*-P4VP)_n_ (n = 1, 2, 3) block copolymer nanoassemblies.

### 2.4. Characterization

The molecular weight (*M*_n_) and dispersity (*Đ*, *Đ* = *M*_w_/*M*_n_) of the polymers were obtained using a Waters 1525 μ gel permeation chromatograph. The samples were passed through three columns using DMF solution containing 0.05 M lithium bromide as the eluent at a flow rate of 1.0 mL/min at 50 °C. The narrow-polydispersity samples of polystyrene were used as a calibration standard. The ^1^H NMR analysis was performed on a Bruker Avance II 400 MHz NMR spectrometer using CDCl_3_ as a solvent. The TEM observation was performed using FEI Tecnai 12 transmission electron microscopy (TEM) with 120 kV or JEM-2100 TEM with 200 kV, whereby a small drop of the block copolymer dispersion was deposited onto a piece of copper grid and then dried at room temperature under vacuum.

## 3. Results and Discussion

### 3.1. Synthesis of macro-CTAs with Linear and Star Structure

**Scheme 1:**
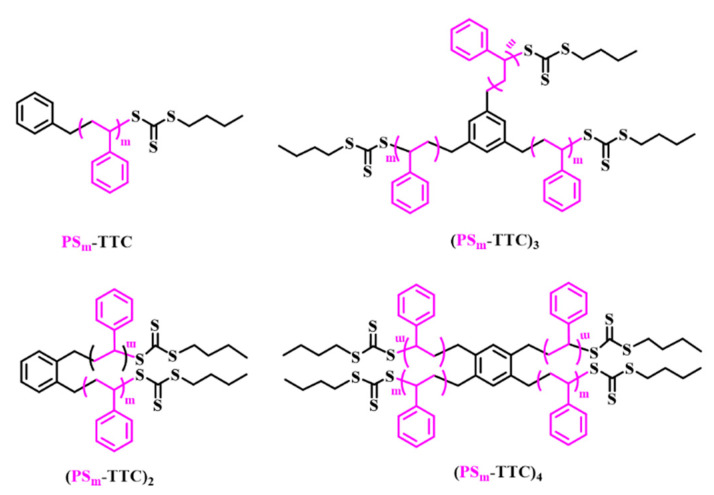
(PS_m_-TTC)_n_ Macro-CTAs.

**Figure 1:**
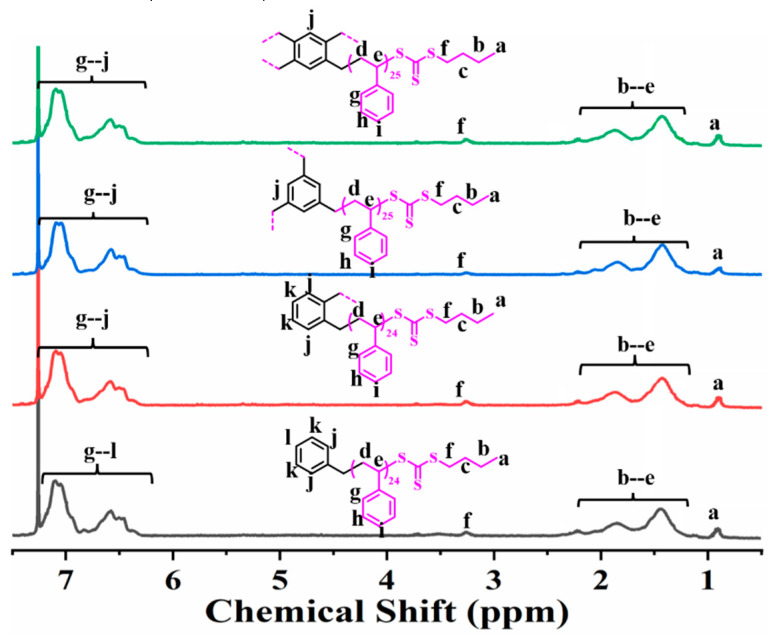
^1^H NMR spectra of (PS-TTC)n (n = 1, 2, 3, 4) with similar PS arms chain at about 25.

**Figure 2:**
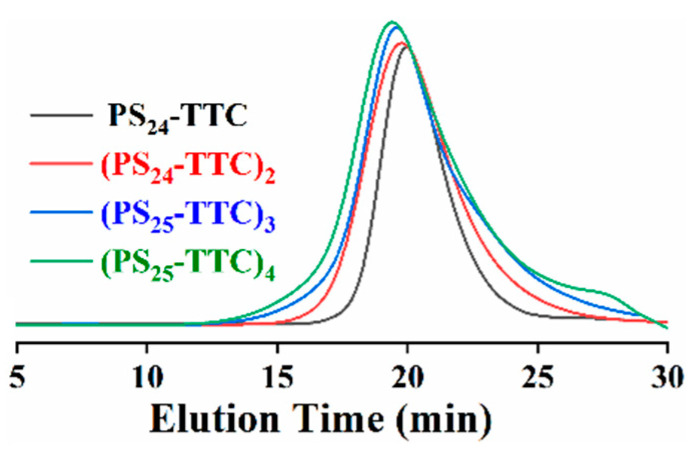
GPC traces of PS-TTC)n (n = 1, 2, 3, 4) with similar PS arms chain at about 25.

Due to the similar R and Z groups in the structure of I, II, III, IV CTAs (Scheme S1), each arm of the synthesized star macro-CTAs was assumed to have a similar degree of polymerization. The linear and star macro-CTAs of (PS)_n_-TTC (n = 1, 2, 3, 4) (Scheme 1) were synthesized by solution RAFT polymerization employing an initiator of AIBN and chain transfer agents (CTAs) of I, II, III or IV. By varying the molar ratio of St/CTAs/AIBN (Table 1), (PS)_n_ with a suitable DP of the PS arms was prepared. The (PS-TTC)_n_ (n = 1, 2, 3, 4) macro-RAFT agents were characterized by ^1^H NMR analysis and GPC analysis, and the results for the typical (PS_24_-TTC)_n_ (n = 1, 2, 3, 4) with a similar DP of the PS arms, around 25, are shown in Figure 1 and Figure 2. The molecular weight, *M*_n,NMR_ by ^1^H NMR analysis was calculated by comparing the integration area of the peaks at 0.88 ppm and 6.20–7.20 ppm attributed to the RAFT terminal and benzene ring. Furthermore, the *M*_n,NMR_ was approximately equal to the theoretical molecular weight *M*_n,th_ calculated from the monomer conversion according to eqn S1 (Table 1).

In Figure 2, unimodal GPC traces were observed for linear PS_24_-TTC, (PS_24_-TTC)_2_, and (PS_25_-TTC)_3_, while for the 4-arm star (PS_25_-TTC)_4_, a small acromion appeared on the high-molecular-weight side. In the synthesis of star polymers, a molecule usually contains more than one propagating radical, which is more likely to lead to a bimolecular termination reaction as discussed elsewhere [44]. However, the dispersity of star (PS-TTC)_n_ was narrow, which can be verified from D < 1.3 (Table 1). It should be noted that the molecular weight obtained by GPC for the star-shaped (PS-TTC)_n_ prepared in this study was close to the amount obtained by NMR characterization, which was not quite consistent with previous results recorded in the literature, in that *M*_n,GPC_ was less than *M*_n,NMR_ for star-shaped polymers [45,46], which will also be discussed in the following sections. (Note: herein the DP values of PS_24_-TTC, (PS_24_-TTC)_2_, (PS_25_-TTC)_3_, and (PS_25_-TTC)_4_ were calculated according to the monomer conversions. In addition, the DP values mentioned in the following sections were calculated through the monomer conversion.)

### 3.2. Effect of the Arm Number on BCP Nanoassemblies Prepared through PISA

It has been reported that the arm number of star polymers has an important effect on the morphology of block copolymer nanoassemblies in ethanol/water. However, the topology of star polymers on the morphology of nanoassemblies formed through PISA in toluene has not been studied. To achieve this, we synthesized a series of (PS-*b*-P4VP)_n_ nanoparticles with different compositions by varying the length of the PS or P4VP chain segments according to Scheme S3, and characterized their morphologies by TEM.

**Figure 3:**
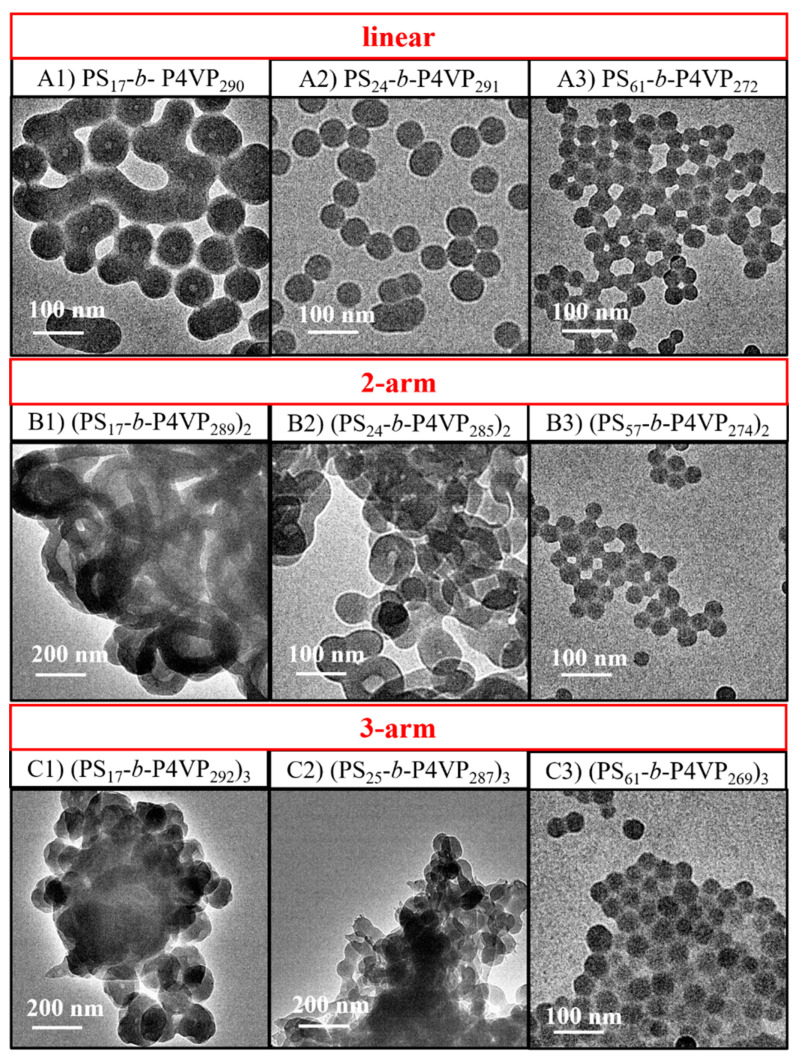

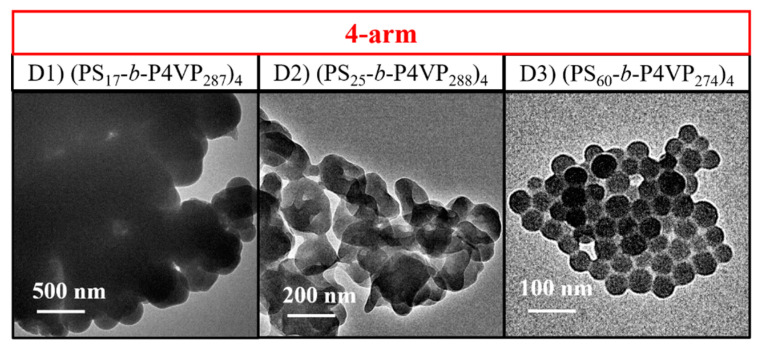
TEM images of (PS-*b*-P4VP)_n_ nanoparticles containing similar P4VP segments but different PS segments.

Figure 3 lists the (PS-*b*-P4VP)_n_ nanoparticles containing similar P4VP segments but different PS segments. The results indicate that all the linear and star (PS-*b*-P4VP)_n_ (n = 1, 2, 3, 4) with the P4VP block chain at about 270 and the long PS arm block chain at about 60 formed discrete nanoassemblies of 32.5 ± 4.6 nm PS_61_-*b*-P4VP_272_ nanospheres, 33.2 ± 2.3 nm (PS_57_-*b*-P4VP_274_)_2_ nanospheres, 40.1 ± 3.6 nm (PS_61_-*b*-P4VP_269_)_3_ nanospheres and 44.3 ± 2.4 nm (PS_60_-*b*-P4VP_274_)_4_ nanospheres, respectively. When the polymerization degree of PS arms is short, such as at about 17 or 24, linear block copolymers still form discrete nanoassemblies, while star block copolymers n = 2, 3, and 4 all formed aggregates, especially the aggregates of the 3- and 4-armed block copolymers. The self-assembly of star block copolymers or linear BAB (B is insoluble chain segment) polymers can also form large aggregates, which may be due to the formation of bridging interactions between nanoparticles [47,48,49,50].

**Figure 4:**
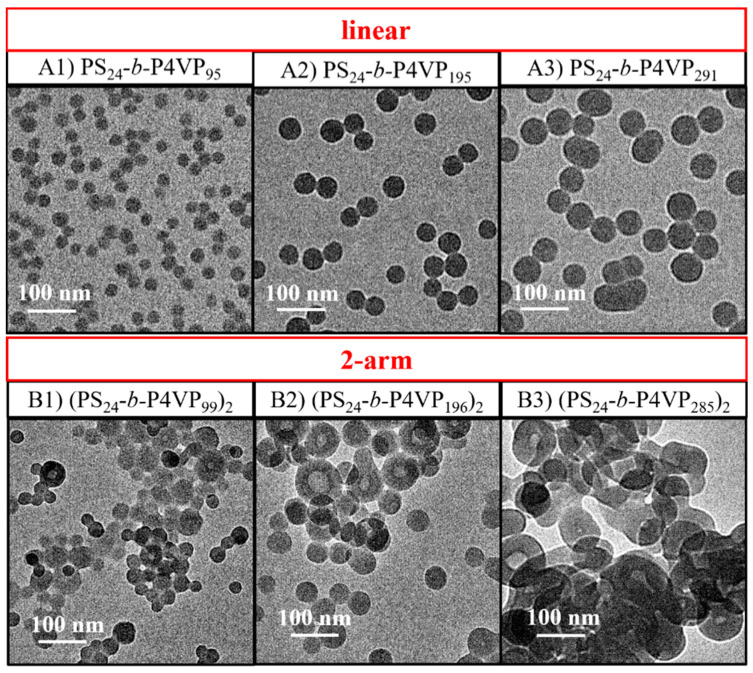

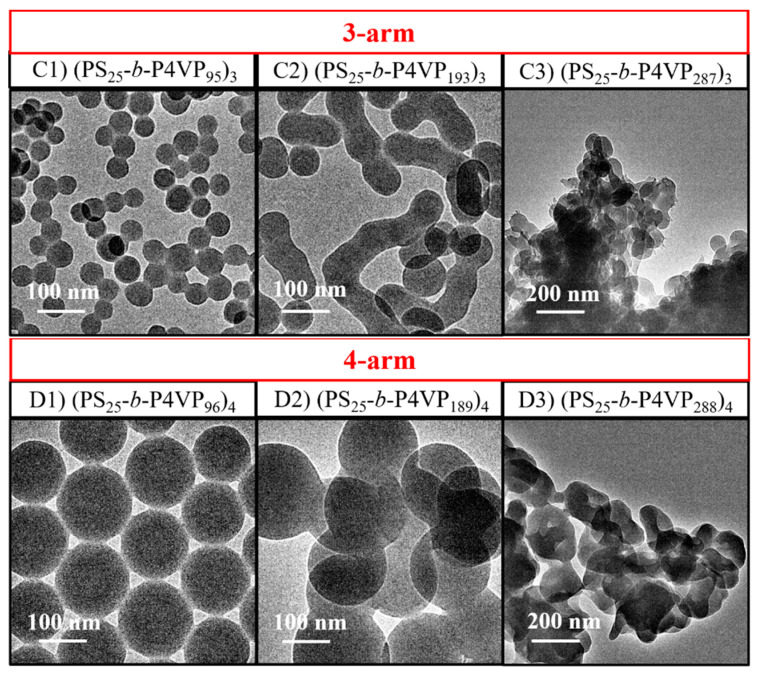
TEM images of (PS-*b*-P4VP)_n_ nanoparticles containing similar PS segments but different P4VP segments.

Figure 4 lists the different case of (PS-*b*-P4VP)_n_ nanoparticles with a similar DP of the PS segments at about 25, while increasing the DP of P4VP segments. Correspondingly, (PS-*b*-P4VP)_n_ (n = 1−4) with a short block chain of P4VP arms (DP < 200) formed discrete nanoassemblies, and star (PS-*b*-P4VP)_n_ (n = 2, 3, 4) with long block chain of P4VP arms at about 285 formed bridged aggregates, while linear PS-*b*-P4VP with long block chain of P4VP still formed discrete nanoassemblies. It was found that both the linear and star (PS-*b*-P4VP)_n_ followed similar rules, i.e., the diameter of their nanoparticles increased with the DP of P4VP block increasing. This rule is consistent with the previous morphologic change in linear BCP nanoparticles [51,52,53,54]. In addition, the morphology of star (PS-*b*-P4VP)_2–3_ nanoparticles is much more complex than that of linear PS-*b*-P4VP.

### 3.3. Effect of the Arm Number on Polymerization Kinetics and the Evolution of (PS-b-P4VP)_n_ Nanoassemblies

To further study the effect of the arm number on the polymerization kinetics and the evolution of (PS-*b*-P4VP)_n_ nanoassemblies, the (PS-TTC)_n_ (n = 1–3) with a similar arm length of about 25 mediated PISA of 4VP was studied in detail. In order to ensure that the polymerization conditions were similar, [4VP]_0_:[trithiocarbonate]_0_:[AIBN]_0_ were designed as a constant in all PISA reactions.

**Figure 5:**
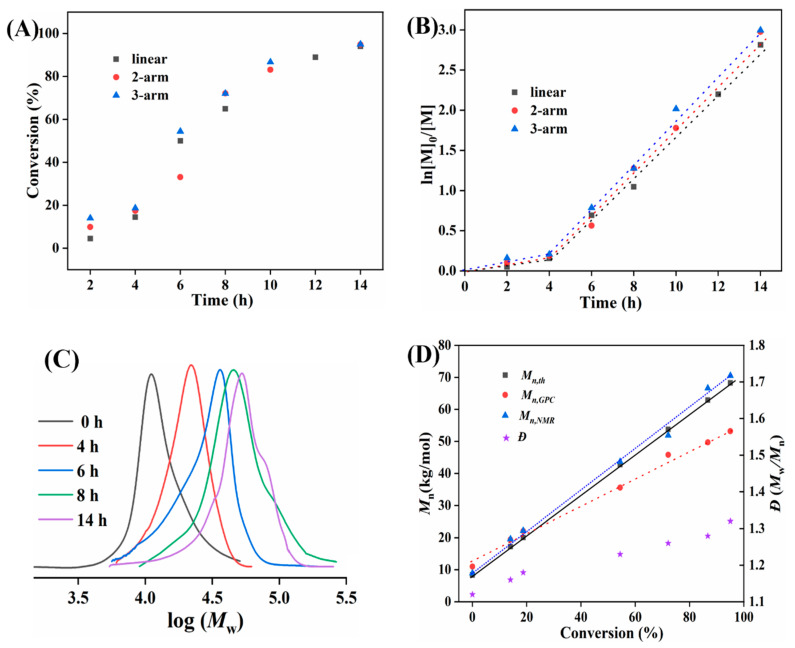
Polymerization kinetics (**A**) and semilogarithmic plots (**B**) of RAFT dispersion polymerization mediated by linear and 2-, and 3-armed star (PS-TTC)_n_. GPC traces (**C**) and evolution of *M*_n,th_, *M*_n,NMR_, *M*_n,GPC_, and *Đ* values (**D**) in (PS_25_-TTC)_3_-mediated PISA of 4VP.

As can be seen in Figure 5A, the monomer conversion-time in the three RAFT dispersion polymerization reactions was very similar, indicating that the three reactions had similar polymerization kinetics. This may be due to the fact that the (PS-TTC)_n_ macromolecular chain transfer agents used had similar R and Z groups and close arm lengths. As shown in Figure 5B, the (PS-TTC)_n_ (n = 1, 2, 3) mediated PISA of 4VP firstly underwent a slow polymerization process, followed by an accelerated polymerization after about 4 h reaction (Figure 5B), which had similar polymerization kinetics as commonly reported for RAFT dispersion polymerization [55,56,57,58,59,60,61]. Generally, the synthesized 3-armed stars (PS_25_-*b*-P4VP)_3_ were characterized by GPC (Figure 5C) and NMR to obtain their *M*_n, GPC_ and *M*_n,NMR_, respectively, and the results are listed in Figure 5D. A small acromion appeared when the monomer conversion was greater than 87% in the GPC curve of (PS_25_-*b*-P4VP)_3_, which was also reflected in the increase in the *Đ* value in Figure 5D. Moreover, the RAFT synthesis was controllable given that (PS_25_-*b*-P4VP)_3_ had a star structure and *Đ* was around 1.3. It is worth mentioning that when monomer conversion was low, the molecular weight obtained by NMR was close to the molecular weight obtained by GPC, which is in agreement with that described above. When the monomer conversion was greater than 20%, the molecular weight obtained by GPC was smaller than that obtained by NMR, which is consistent with previous results [45,46].

**Figure 6:**
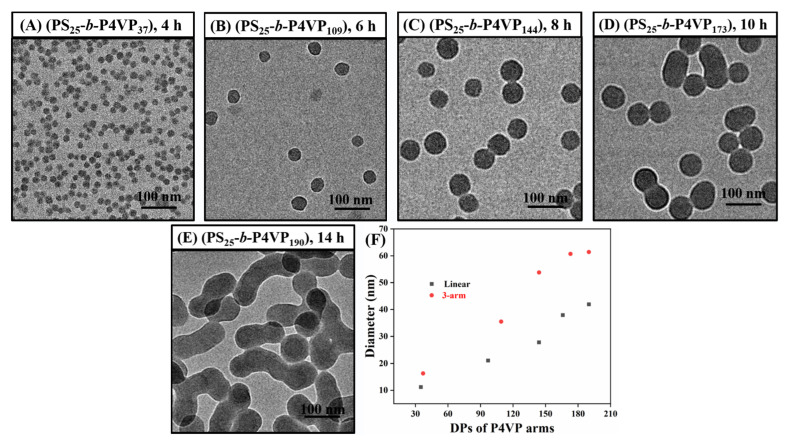
TEM images of 3-armed star (PS_25_-*b*-P4VP)_3_ nanoparticles formed by RAFT dispersion polymerization at 4 (**A**), 6 (**B**), 8 (**C**), 10 (**D**),14 (**E**), and average diameter (*D*) of (PS_25_-*b*-P4VP)_3_ and linear PS_24_-*b*-P4VP nanoparticles with the DP of P4VP arms increasing (**F**).

In addition, the formation process of the 3-armed star-shaped (PS_25_-*b*-P4VP)_3_ nanoparticles was recorded by TEM as shown in Figure 6. With the increase in P4VP, (PS_25_-*b*-P4VP)_3_ nanoparticles changed from 16.3 ± 1.4 nm nanospheres at the beginning of 4 h to 53.8 ± 3.9 nm nanospheres at 8 h, and finally to worms of (PS_25_-*b*-P4VP_190_)_3_. However, the linear PS_24_-*b*-P4VP was still a solid sphere when the length of P4VP was 290 (Figure 4). Figure 6F summarizes the average diameter (*D*) of the (PS_25_-*b*-P4VP)_3_ and linear PS_24_-*b*-P4VP nanoparticles with the DP of P4VP arms increasing, showing that the average diameter of (PS_25_-*b*-P4VP)_3_ changed very little after 10 h, which may be related to the change in morphology from nanospheres to worms. Moreover, the rate of change with the DP was different for the 3-armed (PS_25_-*b*-P4VP)_3_ and linear PS_24_-*b*-P4VP block copolymer nanoparticles.

### 3.4. Effect of the Arm Number on BCP Nanoassemblies Prepared through General Self-Assembly in Toluene

As introduced above, the polymerization-induced self-assembly of block copolymers and the self-assembly of block copolymers in block-selective solvent represent two strategies to prepare block copolymer nano-objects. Herein, the effect of the arm number on BCP nanoassemblies prepared through general self-assembly in toluene was investigated by checking the morphology of the linear and star block copolymer nanoassemblies with similar chemical compositions. To depress the residual monomer effect, the block copolymer nanoassemblies prepared at high monomer conversion through the polymerization-induced self-assembly were chosen. The preparation of the (PS-*b*-P4VP)_n_ (n = 1, 2, 3) block copolymer nanoassemblies through general self-assembly in the block-selective solvent was achieved by initially dissolving the block copolymer in DCM, and then adding toluene slowly, finally removing the excess DCM under vacuum at room temperature as discussed elsewhere [42]. Whereas, differently from the highly concentrated block copolymer in the polymerization-induced self-assembly (~20 wt %), very diluted block copolymer (0.2 mg/mL) was employed in the present self-assembly strategy, since destabilization of the (PS-*b*-P4VP)n (n = 1, 2, 3) block copolymer dispersion was found when the block copolymer concentration was above 1 wt %. Under this diluted block copolymer concentration at 0.2 mg/mL, all the block copolymer nanoassemblies were of great stability.

**Figure 7:**
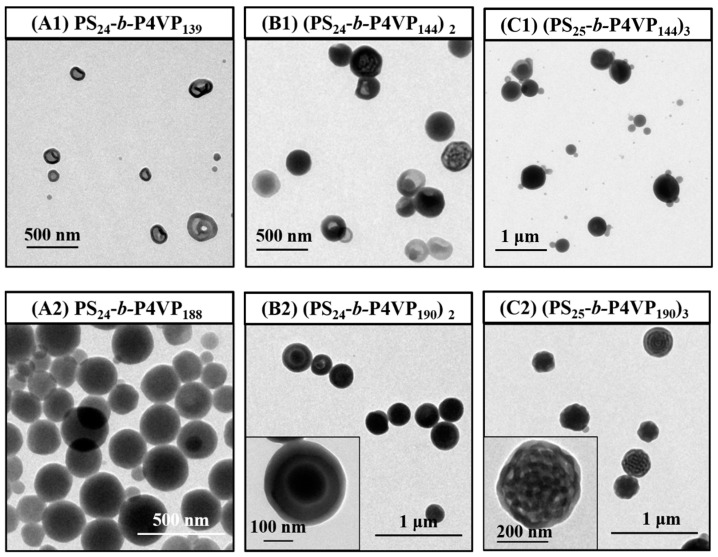
The TEM images of the linear or star block copolymer nanoassemblies of PS_24_-*b*-P4VP_139_ (**A1**) (PS_24_-*b*-P4VP_144_)_2_ (**B1**) (PS_25_-*b*-P4VP_144_)_3_ (**C1**) with similar PS block chains at about 25 and similar P4VP block chains at about 140 (top), and PS_24_-*b*-P4VP_188_ (**A2**), (PS_24_-*b*-P4VP_190_)_2_ (**B2**), (PS_25_-*b*-P4VP_190_)_3_ (**C2**) with similar PS block chains at about 25 and similar P4VP block chains at about 190 (bottom) prepared through general self-assembly in toluene.

Figure 7 shows the TEM images of the linear or star (PS-*b*-P4VP)_3_ block copolymer nanoassemblies with similar PS block chains at about 25 and similar P4VP block chains at about 140 (top in figure) and with similar PS block chains at about 25 and similar P4VP block chains at about 190 (bottom in figure) prepared through general self-assembly in toluene. It indicates that the PS_24_-*b*-P4VP_139_ formed vesicles (Figure 7A1), whereas the similar chemical composition of star block copolymers of (PS_24_-*b*-P4VP_144_)_2_ self-assembled into vesicles and bicontinuous nanospheres (Figure 7B1). Similar bicontinuous nanospheres of the mixture of poly-(ethylene glycol)-*b*-polystyrene/polystyrene-*b*-poly(ethylene glycol)-*b*-polystyrene [62] and poly(ethylene oxide)-*b*-poly(octadecyl methacrylate) containing a long side octadecyl chain [63] and amphiphilic polynorbornene block copolymer [64] were also prepared, and star block copolymers of (PS_25_-*b*-P4VP_144_)_3_ self-assembled into aggregates (Figure 7C1). The self-assembly of PS_24_-*b*-P4VP_188_ in toluene resulted in large nanospheres (Figure 7A2), and the self-assembly of star block copolymers of (PS_24_-*b*-P4VP_190_)_2_ and (PS_25_-*b*-P4VP_190_)_3_ with similar chemical composition lead to multilayered vesicles (Figure 7B2), and bicontinuous nanospheres (Figure 7C2). These results clearly demonstrate that the topology of block copolymers also influenced the nanoassemblies prepared through general self-assembly in toluene.

## 4. Conclusions

In conclusion, linear and star BCP nanoparticles of (PS-*b*-P4VP)_n_ with numbers of 1, 2, 3, and 4 were prepared by two methods of polymerization-induced self-assembly using (PS-TTC)_n_ (n = 1, 2, 3, 4) macro-RAFT agents and self-assembly of block copolymers in the low-polar organic solvent, toluene. Furthermore, the effect of the topology on the size and/or morphology of the (PS-*b*-P4VP)_n_ nanoassemblies synthesized by the two methods in toluene was investigated in detail, and star (PS-*b*-P4VP)_n_ had a more complex morphology than its linear counterpart. In addition, the PISA process of linear or star polymers with different topologies had similar polymerization kinetics. The possible reason is that all the macro-CTAs had similar R and Z groups and similar arm lengths. Our results show that in toluene, a low polar solvent, the topology not only influenced the morphology of the BCP nanoparticles synthesized by PISA, but also affected their nanoassemblies prepared through general self-assembly.

## Figures and Tables

**Table 1:** Summary of the Synthesized Macro-CTAs.

Macro-CTA	[M]_0_:[CTA]_0_:[I]_0_	Time (h)	Conv. *^a^* (%)	*M*_n_ (kg/mol)			*Ð ^e^*
				*M*_n,th_ *^b^*	*M*_n,GPC_ *^c^*	*M*_n,NMR_ *^d^*	
PS_17_-TTC	100:5:1	32	85.0	2.02	3.1	2.1	1.11
PS_24_-TTC	150:5:1	32	80.0	2.75	3.2	3.1	1.11
PS_61_-TTC	500:5:1	32	61.0	6.60	6.4	6.5	1.14
(PS_17_-TTC)_2_	200:5:2	32	85.0	3.97	4.4	4.1	1.12
(PS_24_-TTC)_2_	300:5:2	32	80.4	5.43	5.5	5.38	1.13
(PS_57_-TTC)_2_	1000:5:2	32	57.2	12.29	10.6	12.4	1.18
(PS_17_-TTC)_3_	300:5:3	32	85.0	5.92	5.8	6.0	1.12
(PS_25_-TTC)_3_	450:5:3	32	83.3	8.41	7.9	8.7	1.12
(PS_61_-TTC)_3_	1500:5:3	32	59.8	19.64	21.3	19.5	1.22
(PS_17_-TTC)_4_	400:5:4	32	85.0	7.86	7.8	7.7	1.18
(PS_25_-TTC)_4_	600:5:4	32	83.3	11.19	10.9	11.0	1.20
(PS_60_-TTC)_4_	2000:5:4	32	60.0	25.75	22.6	26.1	1.26

*^a^* Monomer conversion determined by ^1^H NMR. *^b^* Theoretical molecular weight according to eq S1. *^c^* Molecular weight determined by GPC. *^d^* Molecular weight determined by ^1^H NMR. *^e^ Ð* (*M*_w_/*M*_n_) determined by GPC.

## Data Availability

Not applicable.

## Supplementary Materials

The following supporting information can be downloaded at: https://www.mdpi.com/article/10.3390/polym14173691/s1, Scheme S1: Mono- and Multifunctional Trithiocarbonates; Scheme S2: Synthesis of linear and 2, 3, 4-arm CTAs; Figure S1: ^1^H NMR spectra (A) and ^13^C NMR spectra (B) of mono- and multifunctional macro-CTAs of trithiocarbonate. Note: peak e at 220 ppm is out the range of test in the 13 C NMR spectra and Scheme S3: Synthesis of the (PSm-TTC)n (n = 1, 2, 3, 4) macro-RAFT agents and the dispersion RAFT polymerization of 4-vinylpyridine in the presence of (PSm-TTC)n.
