# Supplementary material for: The Effect of Topology on Block Copolymer Nanoparticles: Linear versus Star Block Copolymers in Toluene
Source: Polymers (Basel). 2022 Sep 5;14(17):3691. doi: 10.3390/polym14173691 (PMC9460934; doi:10.3390/polym14173691)
Supplement: Supplementary file 1 [file polymers-14-03691-s001.zip › polymers-1895020-supplementary.pdf]

Supporting Information for:

The effect of topology on the block copolymer nanoassemblies: linear block copolymers versus star block copolymers in toluene

Yuan Zhang,* Peng Wang, Nan Li, Chunyan Guo, Sumin Li

Research School of Polymeric Materials, School of Materials Sciences & Engineering, Jiangsu University, Zhenjiang, 212013, China.

Materials:

Butanethiol (97%), K_3PO_4 (97%) purchased from Sinopharm Chemical Reagent Co. LTD, China, 1,2-bis(bromomethyl)benzene (>98%, Adamas), 1,3,5-tris-(bromomethyl)benzene (97%, Adamas), and 1,2,4,5-tetrakis(bromomethyl)benzene (95%, Adamas) and benzyl bromide (98%, Shanghai chemical reagent, China) were used without any purification. All other chemical reagents were of analytic grade and were commercially available.

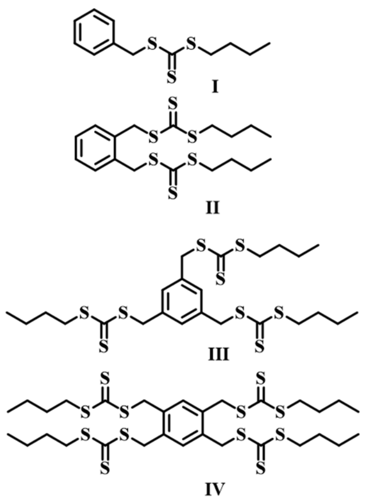

Scheme S1. Mono- and Multifunctional Trithiocarbonates.

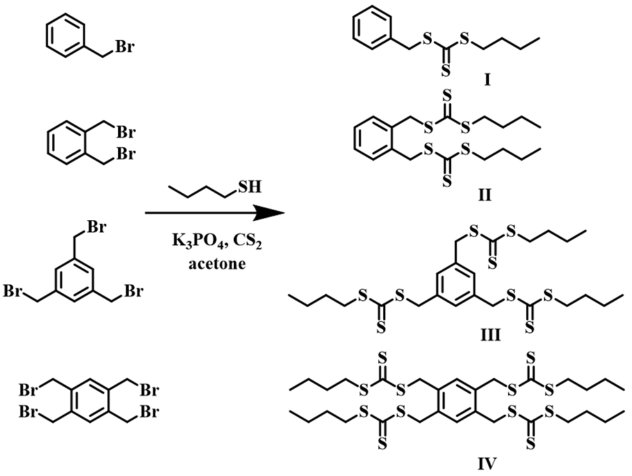

Scheme S2. Synthesis of linear and 2, 3, 4-arm CTAs.

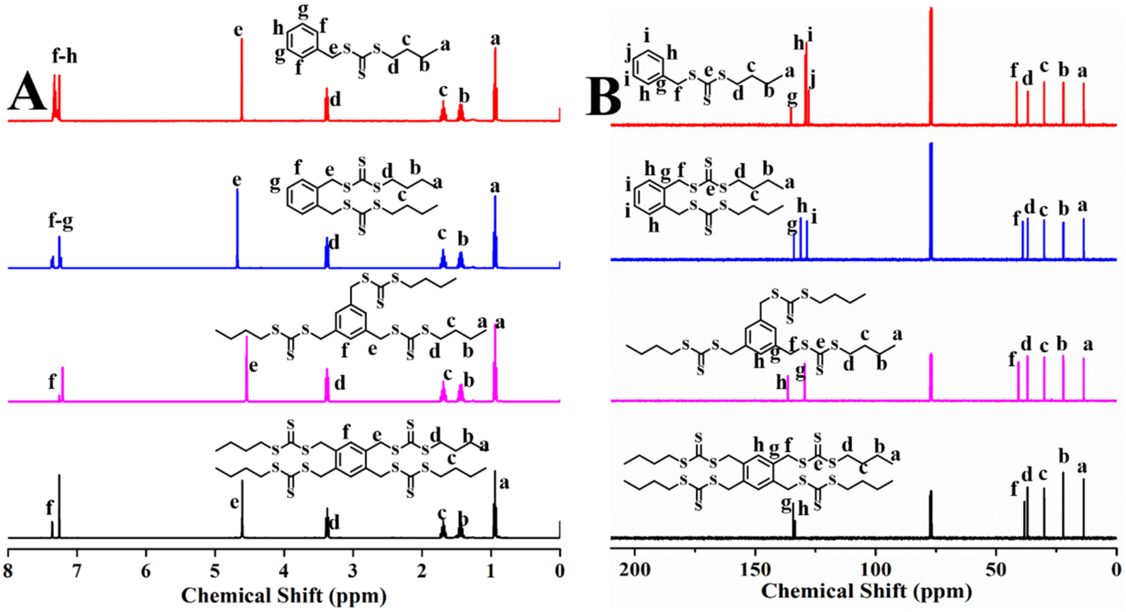

Figure S1. ^{1}H NMR spectra (A) and ^{13}C NMR spectra (B) of mono- and multifunctional macro-CTAs of trithiocarbonate. Note: peak e at 220 ppm is out the range of test in the ^{13}C NMR spectra.

$$M_{n,th} = \frac{[\text{monomer}]_0 \times M_{\text{monomer}}}{[\text{RAFT}]_0} \times Conversion + M_{\text{n,RAFT}} \qquad \text{(eqn S1)}$$

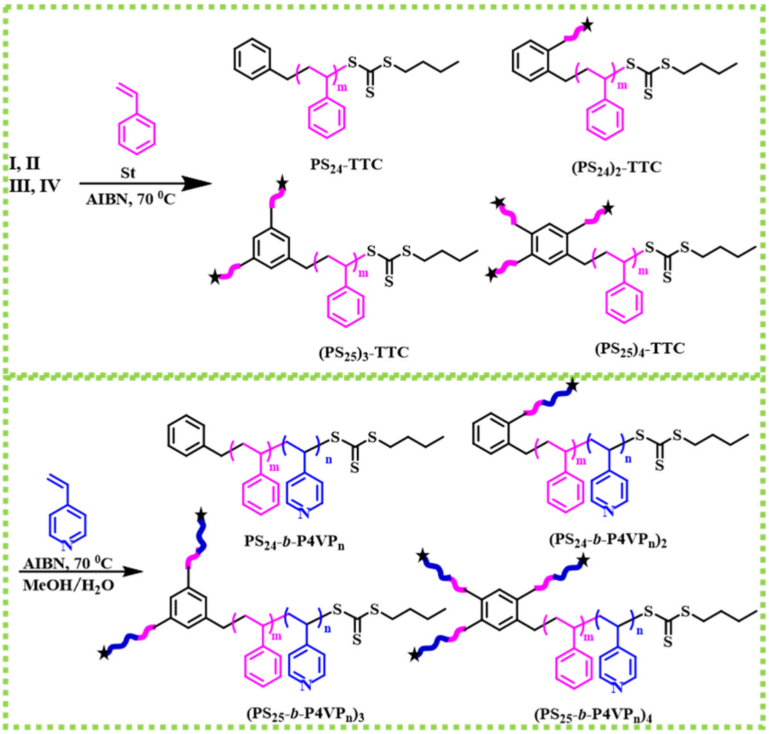

Scheme S3. Synthesis of the $(PS_m\text{-}TTC)_n$ (n = 1, 2, 3, 4) macro-RAFT agents and the dispersion RAFT polymerization of 4-vinylpyridine in the presence of $(PS_m\text{-}TTC)_n$.
